# Zika virus infection reprograms global transcription of host cells to allow sustained infection

**DOI:** 10.1038/emi.2017.9

**Published:** 2017-04-26

**Authors:** Shashi Kant Tiwari, Jason Dang, Yue Qin, Gianluigi Lichinchi, Vikas Bansal, Tariq M Rana

**Affiliations:** 1 Department of Pediatrics, University of California San Diego, La Jolla, CA 92093, USA; 2 Department of Bioengineering, University of California San Diego, La Jolla, CA 92093, USA; 3 Graduate School of Biomedical Sciences, Sanford Burnham Prebys Medical Discovery Institute, 10901 North Torrey Pines Road, La Jolla, CA 92037, USA; 4 Institute for Genomic Medicine, University of California San Diego, La Jolla, CA 92093, USA

**Keywords:** metabolic inhibitor, transcriptome, viral response, Zika virus

## Abstract

Zika virus (ZIKV) is an emerging virus causally linked to neurological disorders, including congenital microcephaly and Guillain–Barré syndrome. There are currently no targeted therapies for ZIKV infection. To identify novel antiviral targets and to elucidate the mechanisms by which ZIKV exploits the host cell machinery to support sustained replication, we analyzed the transcriptomic landscape of human microglia, fibroblast, embryonic kidney and monocyte-derived macrophage cell lines before and after ZIKV infection. The four cell types differed in their susceptibility to ZIKV infection, consistent with differences in their expression of viral response genes before infection. Clustering and network analyses of genes differentially expressed after ZIKV infection revealed changes related to the adaptive immune system, angiogenesis and host metabolic processes that are conducive to sustained viral production. Genes related to the adaptive immune response were downregulated in microglia cells, suggesting that ZIKV effectively evades the immune response after reaching the central nervous system. Like other viruses, ZIKV diverts host cell resources and reprograms the metabolic machinery to support RNA metabolism, ATP production and glycolysis. Consistent with these transcriptomic analyses, nucleoside metabolic inhibitors abrogated ZIKV replication in microglia cells.

## INTRODUCTION

Zika virus (ZIKV) is an emerging arbovirus of the Flaviviridae family,^1, 2^ which includes West Nile (WNV), yellow fever, chikungunya, dengue and Japanese encephalitis viruses.^2^ These viruses cause mosquito-borne diseases transmitted by the *Aedes* genus.^2^ ZIKV may also be transmitted sexually and vertically.^3, 4^ ZIKV was first discovered >60 years ago in samples taken from a sentinel rhesus monkey in the Zika forest of Uganda, and has since been isolated from mosquitoes and humans.^5, 6^ Various epidemiological studies have revealed a worldwide spread of ZIKV to geographic areas ranging from Asia and the Pacific to, most recently, the Americas.^1^ The rapid spread of ZIKV from Asia to the Americas has affected >30 countries. Due to its sporadic nature and mild symptoms, ZIKV infection was initially ignored. Approximately 80% of ZIKV infections are asymptomatic, and the most common symptoms include fever, arthralgia, rash, myalgia, edema, vomiting and non-purulent conjunctivitis.^7^ However, ZIKV infection in pregnant women has been linked to the increasing incidence of congenital microcephaly and other disorders such as placental insufficiency, fetal growth retardation and fetal death. Emerging evidence suggests that ZIKV causes mild symptoms in non-pregnant individuals, but it has also been associated with neurological abnormalities and Guillain–Barré syndrome.^8, 9, 10, 11^

Female *Aedes* mosquitoes act as vectors to transmit ZIKV through the skin of the mammalian host, which is followed by infection of permissive cells through specific receptors. Current reports indicate that dermal fibroblasts, dendritic cells, neural progenitor cells and epidermal keratinocytes are permissive to ZIKV infection, whereas placental trophoblasts are resistant due to constitutive release of type III interferon.^12, 13, 14, 15, 16^ Interferon knockout mouse models have also shown susceptibility to ZIKV infection.^17, 18, 19^ However, the pathogenesis of ZIKV infection remains poorly understood. In this study, we analyzed transcriptomic changes induced by ZIKV infection in four human cell lines (microglia, fibroblast, macrophage and human embryonic kidney cells) to identify genes that could be developed as potential therapeutic targets and to provide insight into the interaction between ZIKV and the host cell.

## MATERIALS AND METHODS

### Cell lines and culture conditions

Vero, microglia, THP-1, BJ and 293FT cells were maintained under standard culture conditions at 37 °C in a 5% CO_2_ atmosphere. In brief, Vero cells, derived from African green monkey kidney cells, were maintained in Eagle's minimum essential medium supplemented with 10% (vol/vol) fetal bovine serum (FBS) and antibiotics. THP-1 cells, a human leukemia monocytic cell line, were cultured in RPMI 1640 medium supplemented with 10% FBS and 50 μM β-mercaptoethanol (Sigma, St Louis, MO, USA). THP-1 cells were differentiated into macrophages by treatment with 5 ng/mL phorbol-12-myristate-13-acetate (PMA) overnight. The following day, the medium was replaced with fresh medium without PMA. 293FT human embryonic kidney cells and the human fibroblast cell line BJ were cultured in Dulbecco's modified Eagle's medium (Invitrogen, Carlsbad, CA, USA) supplemented with 10% FBS. The human microglial cell line (kindly provided by Dr Jon Karn lab at the Case Western Reserve University, Cleveland, OH, USA) was cultured in Dulbecco's modified Eagle's medium with high-glucose supplemented with 10% FBS and 1% penicillin/streptomycin.

### ZIKV propagation and infection of cell lines

ZIKV prototype MR766 was propagated in the low-passage Vero cell line. Vero cells were infected with virus at a multiplicity of infection (MOI) of 1 in Eagle's minimum essential medium supplemented with 10% FBS. The medium was replaced with fresh medium 24 h after infection, and the viral supernatant was collected at 48 h post infection. Viral titers were assessed using iScript One-Step RT-PCR kit (Bio-Rad, Hercules, CA, USA), and the viral copy number was calculated from a standard curve of *in vitro* transcribed viral RNA transcripts. For infection, cell lines were seeded in six-well culture plates at a density of 1 × 10^6^ cells per well. ZIKV, diluted to the desired MOI (1), was added to the cells and the plates were incubated at 37 °C in a 5% CO_2_ atmosphere for 6, 12, 24 or 48 h. As controls, cells were incubated with culture supernatants from uninfected Vero cells (mock-infected controls). At the indicated times post infection, cell supernatants were collected for determination of viral copy number.

### Immunofluorescence microscopy

To assess ZIKV infection, cells were collected at 24 h following infection and immunostained as described previously.^16^ In brief, ZIKV- and mock-infected cells were fixed with 4% paraformaldehyde in phosphate-buffered saline (PBS) for 20 min at room temperature. Cells were blocked by incubation in 3% BSA and 0.1% Triton X-100 for 2 h at room temperature and then incubated overnight at 4 °C with ZIKVE/anti-flavivirus group antigen antibody (1:500, mouse, Millipore, Temecula, CA, USA; MAB10216), which is directed against the flavivirus envelope protein. Cells were washed with PBS and incubated for 2 h at room temperature with fluorescein isothiocyanate-conjugated anti-mouse IgG. The nuclei were stained using Hoechst 33258 (Invitrogen). Immunostained cells were imaged using a Leica fluorescence microscope (DMI 3000B) (Leica Microsystem, Wetzlar, Germany).

### RNA extraction, cDNA synthesis and qRT-PCR

For cellular messenger RNA (mRNA) analysis, RNA was extracted from the cell lines using an RNeasy Mini kit (Qiagen, Hilden, Germany), following the manufacturer's instructions. RNA samples were treated with RNase-free DNase (Qiagen) and complementary DNA (cDNA) was generated from total RNA (500 ng per sample) using iScript Mastermix (Bio-Rad), according to the manufacturer's instructions. Quantitative PCR (qPCR) was performed with SYBR Green PCR MasterMix (Bio-Rad) using a Roche LightCycler 480(Roche Diagnostics, Basel, Switzerland).

### RNA-Seq and data analysis

For RNA-seq analysis, RNA was extracted from the cell lines using an RNeasy Mini kit (Qiagen), following the manufacturer's instructions. RNA was ribo-depleted, and RNA sequencing was performed using an Illumina NextSeq 500 (Illumina, San Diego, CA, USA) with an average of 20 million reads per sample at The Scripps Research Institute NGS Core facility. The single-end reads that passed Illumina filters were filtered for reads aligning to transfer RNA, ribosomal RNA, adapter sequences and spike-in controls. The reads were then aligned to UCSC hg19 reference genome using TopHat (v 1.4.1, Baltimore, MD, USA). DUST scores were calculated with PRINSEQ Lite (v 0.20.3, open source software), and low-complexity reads (DUST>4) were removed from the BAM files. The alignment results were parsed via the SAMtools to generate SAM files. Read counts to each genomic feature were obtained with the htseq-count program (v 0.6.0) using the ‘union' option. After removing absent features (zero counts in all samples), the raw counts were imported into R/Bioconductor package DESeq2 to identify differentially expressed genes among the samples. DESeq2 normalizes counts by dividing each column of the count table (samples) by the size factor of this column. The size factor is calculated by dividing the samples by the geometric means of the genes. This brings the count values to a common scale suitable for comparison. *P*-values for differential expression were calculated using binomial test for differences between the base means of two conditions. The *P*-values were adjusted for multiple test correction using the Benjamini–Hochberg algorithm to control the false discovery rate. Cluster analyses, including principal component analysis and hierarchical clustering, were performed using standard algorithms and metrics. Gene ontology analyses on biological processes were performed using The Database for Annotation, Visualization and Integrated Discovery.^20^ Grouped functional pathway/gene ontology network analyses were performed using Cytoscape with the ClueGo add-on.^21, 22^

### Drug treatment

Human microglial cell line was infected with ZIKV at MOI of 1 in cell medium containing metabolic inhibitors such as 5-fluorouracil (ab142387; Abcam, Cambridge, UK) and floxuridine (4659; Tocris, Bristol, UK) in cell medium containing 1 and 10 μM of each drug or 1% (vol/vol) dimethylsulfoxide (DMSO) as a control. After 48 h post infection, cellular RNA was extracted using Trizol and cDNA was synthesized by iScript Mastermix (Bio-Rad), as per the manufacturer's instructions. ZIKV RNA was quantified by using specific primers by SYBR Green PCR Master Mix (Bio-Rad), using a Roche LightCycler 480. Further, immunocytochemistry was also performed in microglial cells for the detection of ZIKV infection using flavivirus group antigen-specific antibody (Millipore).

## RESULTS

### Cell-type-specific ZIKV replication and infection

**Figure 1:**
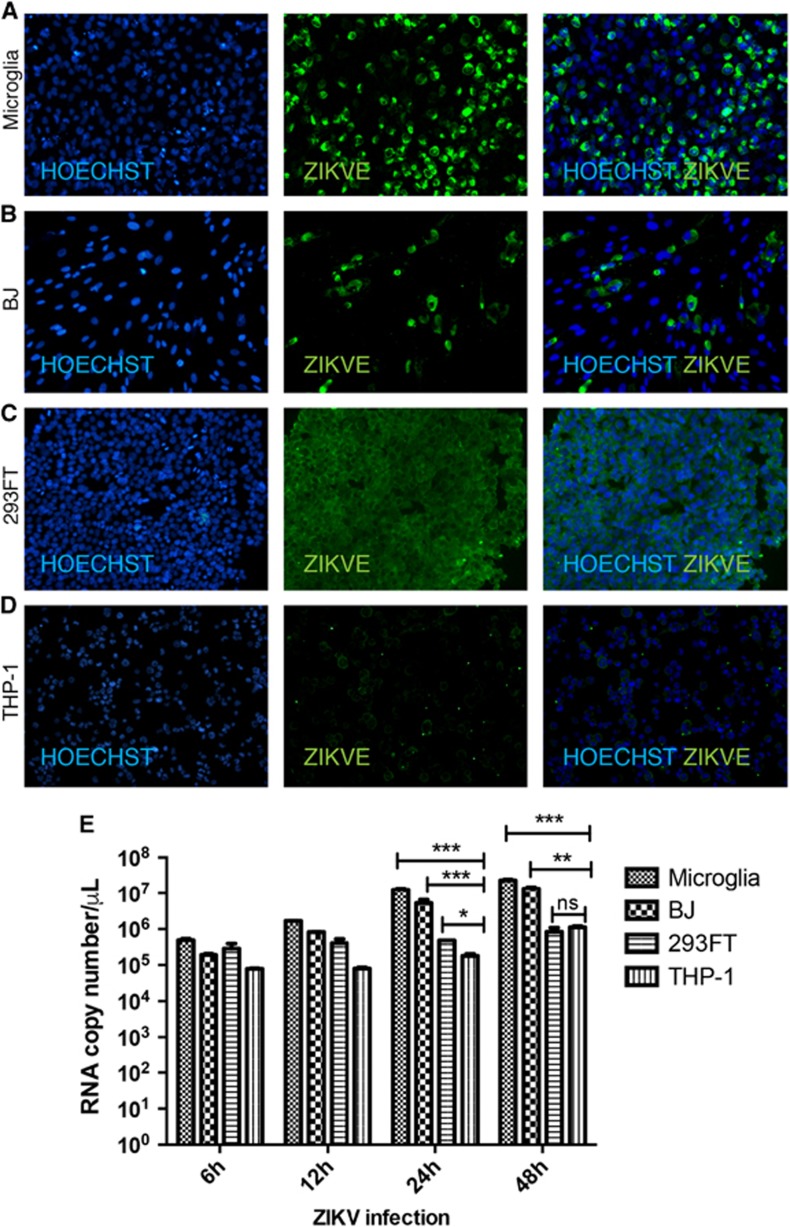
Variable ZIKV infection and replication levels in different cell types. (**A**–**D**) Immunohistochemistry of ZIKV gene expression in microglial, fibroblast (BJ), kidney (293FT) and macrophage (THP-1) cell lines at 24 hpi. Cells were stained with anti-flavivirus envelope protein (ZIKVE), and nuclei were visualized with Hoechst 33258 (Invitrogen). (**E**) ZIKV replication assessed by one-step quantitative PCR with reverse transcription analysis of viral supernatants at the indicated times post infection. Data are presented as the mean±SEM of *n*=3. *P<0.05, **P<0.01, ***P<0.001; ns, not significant by two-way analysis of variance. See also Supplementary Figure S1. Zika virus, ZIKV.

To analyze factors contributing to ZIKV pathogenesis, we selected four human cell lines, microglia, BJ (foreskin fibroblast), 293FT (embryonic kidney) and THP-1-derived macrophages (monocyte-derived macrophage), and inoculated them with ZIKV produced in Vero and BHK cells at a MOI of 1 (Supplementary Figure S1). The choice of cell lines was driven by a desire to understand different aspects of ZIKV pathogenesis. Microglia cells model the resident macrophages in the brain and provide information on how they may contribute to neuroinflammation and other ZIKV-associated neurodegenerative disorders, such as Guillain–Barré syndrome. BJ cells were selected to model dermal infection, the primary route of mosquito-driven ZIKV infection. The 293FT human embryonic kidney cell line is well known for robust lentivirus production and may provide an ideal host for ZIKV replication as well. Last, THP-1 macrophages provide a model of the effect of ZIKV infection on a host cell critical for the immune response. ZIKV expression was assessed by immunofluorescent staining of cells with an antibody against the ZIKVE flavivirus envelope protein at 24 h post infection (hpi). ZIKV infection was most marked in microglia cells, followed by BJ, 293FT and THP-1-derived macrophages (Figures 1A–1D). These findings were confirmed by one-step qPCR with reverse transcription (RT-qPCR) of viral RNA in the cell supernatants, which showed statistically significant differences in the levels of viral transcripts between microglia and THP-1-derived macrophages at 24 and 48 hpi (Figure 1E). This marked difference in ZIKV susceptibility is notable because both cell types are macrophages; microglia cells are microglial and THP-1-derived macrophages are monocyte-derived.

**Figure 2:**
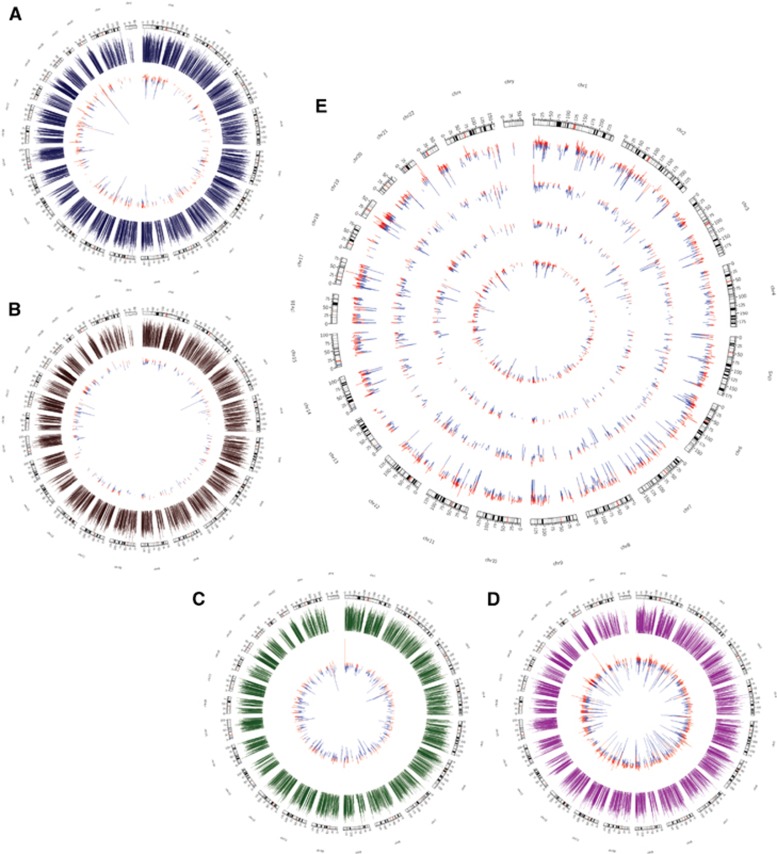
Changes in the transcriptional landscape following ZIKV infection. (**A**–**D**) Circos plots of transcriptional changes in microglia (**A**), BJ (**B**), 293FT (**C**) and THP-1-derived macrophages (**D**) at 24 hpi. The outer circles (blue, brown, green and magenta) represent the expression levels of the transcripts before infection. The inner circles represent the differentially expressed genes, with the size of the lines indicating the fold change in expression. Genes upregulated and downregulated by ZIKV infection are shown in red and blue, respectively. (**E**) Circos plot showing differentially expressed genes in all four cell types. The number of differentially expressed genes and the magnitude of the expression change are inversely correlated with ZIKV expression. Upregulated genes are in red and downregulated genes are in blue. Outer to inner circles: THP-1, 293FT, BJ and microglia. Zika virus, ZIKV.

We next analyzed changes in the transcriptomic landscapes of the four cell types after ZIKV infection to identify potential key regulators responsible for the cell-type-specific differences in ZIKV replication. For this, total RNA was extracted from mock- and ZIKV-infected cell lines at 24 hpi and analyzed by Illumina NextSeq 500. We elected to examine gene expression at 24 hpi to assess the effects of ZIKV infection immediately following the early innate immune response (Supplementary Figures S1C–S1E). Figure 2 shows the data presented in Circos plots, in which the fold change for differentially expressed genes (shown in inner circles) are plotted in contrast with the overall gene expression level (in outer circles) for microglia, BJ, 293FT and THP-1-derived macrophages (Figures 2A–2D, respectively). Interestingly, the level of ZIKV expression and the impact of ZIKV infection on the transcriptome were inversely correlated. Thus, microglia cells showed the fewest changes in gene expression following infection (Figure 2A), followed by BJ (Figure 2B), 293FT (Figure 2C) and THP-1-derived macrophages (Figure 2D). Furthermore, the magnitude of the gene expression changes also differed between the cell types, with the differentially expressed genes in THP-1-derived macrophages displaying the biggest differences between mock- and ZIKV-infected cells (Figure 2E).

### Analysis of viral response genes identifies potential cell-type-specific regulators

**Figure 3:**
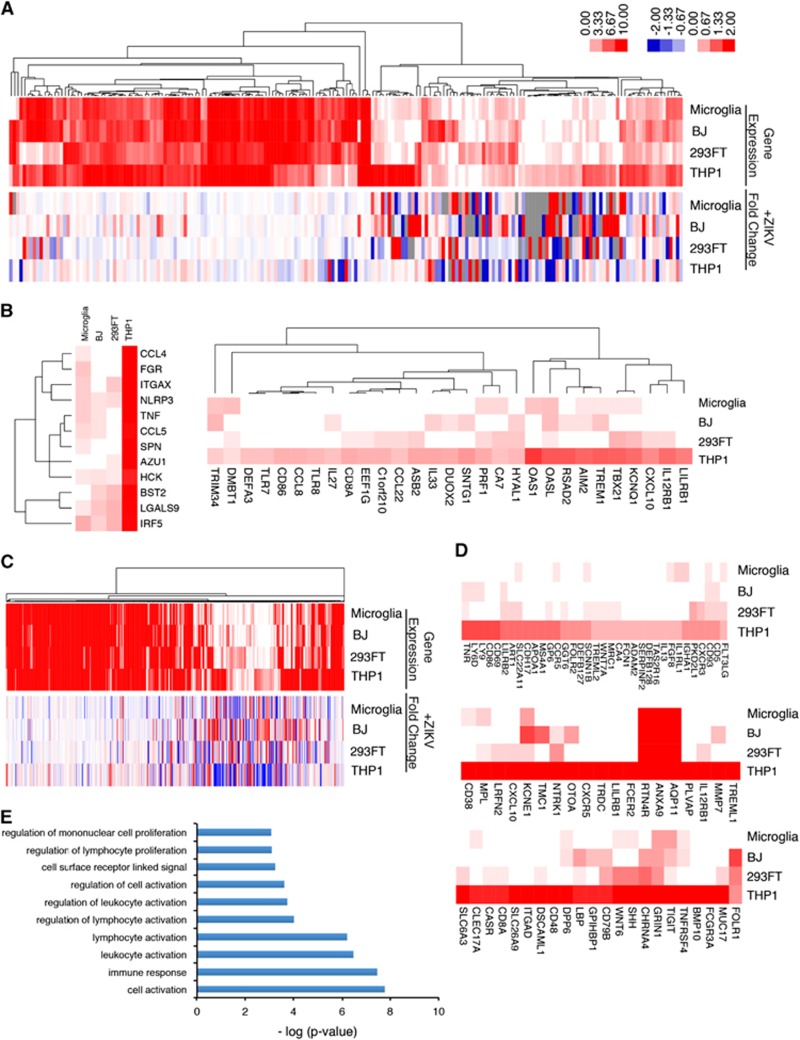
Cell-type-specific differences in steady-state expression of viral response genes reveal potential antiviral targets. (**A**) Hierarchical clustering of genes associated with ‘response to virus' (top) and the fold change in expression of the same genes at 24 hpi (bottom). Left scale bar: 0:10 represents gene expression log_2_ (RPKM+1) for all cell lines. Right scale bar: −2:2 represents fold change in gene expressed between mock-treated and ZIKV-infected cells. Also see Supplementary Table S1. (**B**) Pre-infection expression levels of genes associated with ‘response to virus.' (**C**) Hierarchical clustering of genes associated with ‘cell surface' (top) and their associated fold change in expression following infection (bottom). Also see Supplementary Table S3. (**D**) Differential gene expression of cell surface proteins and receptors involved in cell activation, immune response and cell surface signaling in THP-1-derived macrophages. See also Supplementary Figure S2. (**E**) Gene ontology analysis of cell surface genes highly expressed specifically in THP-1. Zika virus, ZIKV.

To identify genes that may account for the different levels of ZIKV expression in the four cell lines, we analyzed the expression of endogenous viral response genes before infection. We hypothesized that the initial ZIKV viral entry and replication mechanisms might be dependent on the expression levels of specific host genes. Thus, genes from the human GRCh38.p5 database in Ensembl associated with the following gene ontology terms were analyzed in the mock-infected cells: ‘response to virus,' ‘modulation of virus host gene expression,' ‘viral transcription' and ‘viral release from host cell' (Figures 3A and 3B; Supplementary Figures S2A–S2D; Supplementary Table S1).

Consistent with our hypothesis, THP-1-derived macrophages, which were the most resistant to ZIKV infection, expressed the greatest number of viral response genes and immune-related genes, whereas microglia cells express the fewest (Supplementary Figures S2E and S2F; Supplementary Table S2). Genes that were uniquely or highly expressed in THP-1-derived macrophages but showed low expression in microglia were analyzed as potential antiviral ZIKV factors. Interestingly, expression of immune regulatory molecules, including *CCL3*, *CCL4*, *CCL5*, *TNF*, *IRF5*, *CXCL10*, *OAS1*, *TLR7*, *TLR8*, and *IL27*, was highest in THP-1-derived macrophages, indicating that they are primed to mount a vigorous defense as part of the initial innate immune response to ZIKV and to elicit greater transcriptional changes in response to ZIKV infection (Figures 2E and 3B). Comparison of genes differentially expressed in the four cell types before infection revealed high expression of *CD4* in THP-1-derived macrophages and low expression of *CHMP4C* (charged multivesicular body protein 4C). CHMP4C is a component of the ESCRT-III family responsible for surface receptor degradation and viral budding.^23^ Further mechanistic studies will be necessary to determine which of these viral response and immune-related genes might be vital for regulating ZIKV expression.

We also examined the differential expression of cell surface receptor genes in the four cell types prior to ZIKV infection to identify potential antiviral receptors that may confer greater ZIKV resistance on THP-1-derived macrophages. Heat map clustering of cell surface receptor genes identified several genes that were expressed highly in THP-1 compared with the other cell types, including *CD86*, *LY6D*, *CXCL10*, *CD48* and *IL12RB1* (Figures 3C and 3D; Supplementary Table S3). Gene ontology analysis of these and other genes showing selectively high expression in THP-1-derived macrophages revealed an enrichment of genes involved in cell activation, immune response and cell receptor signaling (Figure 3E). These analyses of the steady-state expression of endogenous genes in the four cell lines before ZIKV infection have identified a number of antiviral genes highly expressed in THP-1-derived macrophages, suggesting that they could be exploited to therapeutic effect. As each cell line expresses specific sets of genes that are unique to that cell type, a correlation between steady-state expression of viral response genes and viral susceptibility may not imply a causal relationship and further functional validation is necessary to confirm a role in ZIKV infection.

### ZIKV infection modulates the metabolic and transcriptional landscape

**Figure 4:**
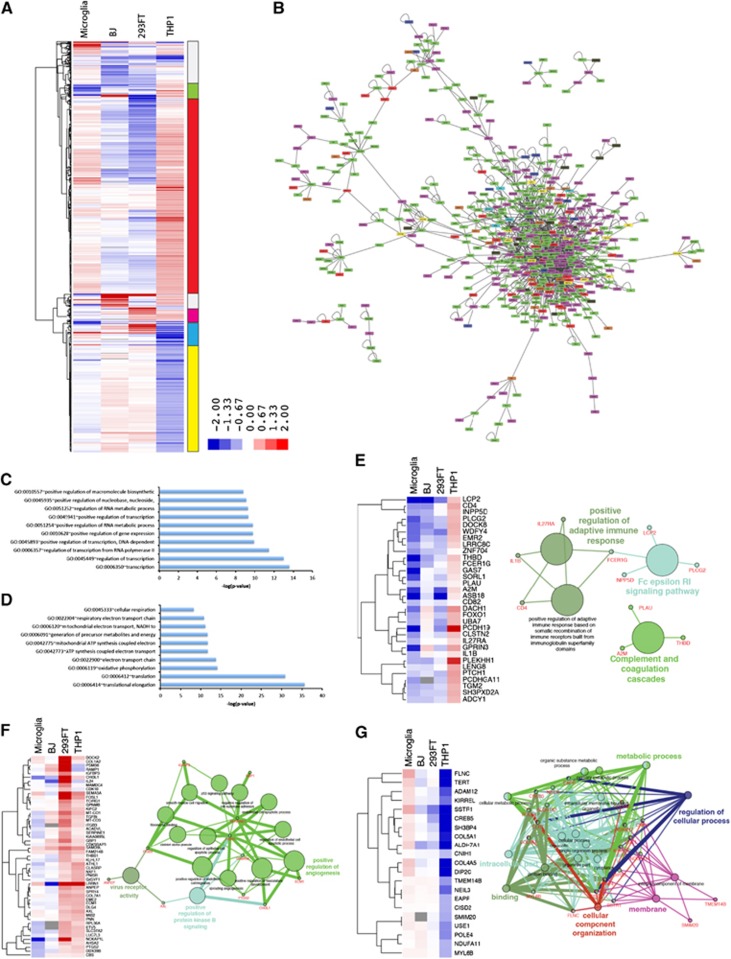
Analysis of differentially expressed genes post ZIKV infection identifies key pathways exploited by ZIKV. (**A**) Hierarchical clustering of differentially expressed genes following ZIKV infection, showing cell-type-specific transcriptional changes. Genes displayed have fold changes of >1.4 and *P*<0.05 in at least one cell type. The vertical bar to the right indicates the differentially expressed gene clusters described by color in the text. Also see Supplementary Table S4. (**B**) Interactome of differentially expressed genes across all cell types. (**C**) Gene ontology analysis of ‘red cluster' genes, which are upregulated in microglia and THP-1-derived macrophages. (**D**) Gene ontology analysis of ‘yellow cluster' genes, which are mostly downregulated in microglia and THP-1-derived macrophages. (**E**) Analysis of ‘green cluster' genes, which are upregulated only in THP-1. (**F**) Analysis of ‘magenta cluster' genes, which are upregulated in 293FT and THP-1-derived macrophages. (**G**) Analysis of ‘blue cluster' genes, which are only upregulated in microglia cells. See also Supplementary Figure S3. Zika virus, ZIKV.

To determine the effects of ZIKV infection on the host transcriptome, we analyzed differentially expressed genes between mock- and ZIKV-infected cell lines at 24 hpi. As mentioned above, the total number of differentially expressed genes in ZIKV-infected cells was inversely correlated with the level of ZIKV infection, with the least- and most-marked changes occurring in microglia and THP-1-derived macrophages, respectively (Figures 1 and 2E). In-depth analysis of the differentially expressed genes revealed that ZIKV infection elicited a similar pattern of gene expression changes between BJ and 293FT cells, and between microglia and THP-1-derived macrophages, both macrophage cell types; however, the fold change in expression is much greater in THP-1-derived macrophages (Figure 4A; Supplementary Table S4). Predictive network analyses suggested that a significant portion of the differentially expressed genes following ZIKV infection interact to regulate common pathways such as including mRNA processing, metabolic processes, RNA processing and cellular component disassembly (Figure 4B; Supplementary Figures S3A–S3D).

Further, clustering analysis highlighted the distinct patterns of gene expression changes in the ZIKV-infected cells (right vertical bar in Figure 4A). Analysis of genes that were predominantly upregulated in microglia and THP-1 macrophages, and downregulated in BJ and 293FT cells (red on the vertical bar in Figure 4A) indicated that they are associated with transcriptional regulators, RNA metabolic processes and macromolecule biosynthesis (Figure 4C). Genes upregulated in THP-1 macrophages and downregulated in all other cell lines (green bar) included *IL1B*, *CD4*, *IL27RA* and *FCER1G* which act as positive regulators of the adaptive immune response, Fc epsilon RI signaling pathway, and complement and coagulation cascades (Figures 4A and 4E). Interestingly, these genes are downregulated in microglia, BJ and 293FT. These findings help to explain why THP-1-derived macrophages, which have the highest expression of innate immune genes in the uninfected state and the greatest upregulation of adaptive immune response genes post infection, have the lowest level of ZIKV expression.

The cluster of genes downregulated by ZIKV infection in microglia and BJ but upregulated in 293FT and THP-1-derived macrophages (magenta bar, Figure 4A) included several key genes that regulate virus receptor activity, protein kinase B activity and angiogenesis (Figure 4F). A notable gene in this cluster is the viral entry receptor AXL, which has previously been associated with ZIKV pathogenesis.^12, 24^ In contrast, the gene clusters downregulated in the macrophage lines (yellow bar in Figure 4A), and particularly in THP-1-derived macrophages, are strongly associated with translational elongation and various cellular metabolic processes (Figure 4D). Finally, genes differentially expressed in ZIKV-infected microglia and THP-1-derived macrophages (blue bar in Figure 4A) were related to metabolic processes (Figure 4G). Viruses are obligate parasites that exploit the host's metabolic processes to reproduce.^25, 26, 27^ Understanding the mechanisms by which ZIKV alters the host cell metabolism may thus provide additional novel therapeutic targets. Further examination of the roles of metabolic control genes such as *TERT*, *ALDH7A1*, *CREB5*, *EAPP*, and *NDUFA11* as they relate to ZIKV infection may provide further insights into ZIKV pathogenesis.

**Figure 5:**
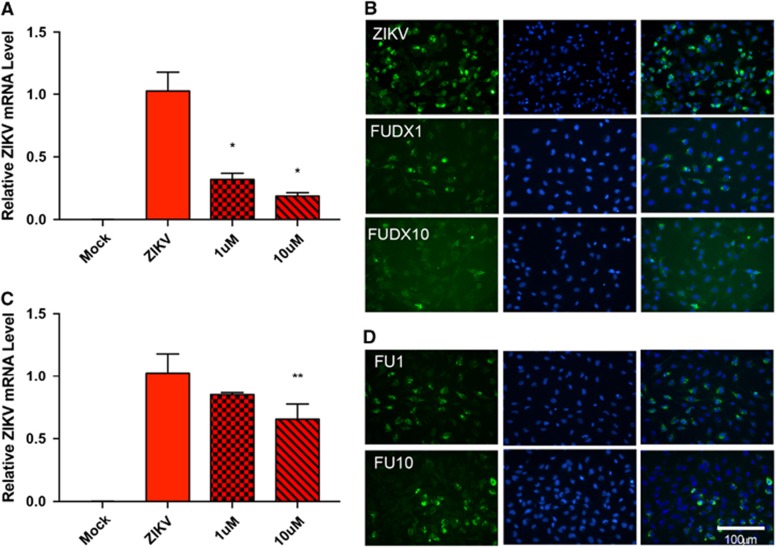
Nucleoside metabolic inhibitors attenuate ZIKV replication. (**A**) ZIKV replication assessed by RT-qPCR 48 hpi in mock, ZIKV only, ZIKV plus 1 μM floxuridine (FUDX1) or ZIKV plus 10 μM floxuridine (FUDX10)-treated microglia. Data are presented as the mean±sem of *n*=3. *P<0.05, **P<0.01 by *t*-test. (**B**) Immunohistochemistry of anti-flavivirus envelope protein (ZIKVE) expression in mock, ZIKV only, ZIKV plus FUDX1 or ZIKV plus FUDX10-treated microglia. (**C**) ZIKV replication assessed by RT-qPCR analysis 48 hpi in mock, ZIKV only, ZIKV plus 1 μM flurouracil (FU1) or ZIKV plus 10 μM flurouracil (FU10)-treated microglia. Data are presented as the mean±SEM of *n*=3. *P<0.05, **P<0.01 by *t*-test. (**D**) Immunohistochemistry of ZIKVE expression in mock, ZIKV only, ZIKV plus 1 μM FU1 or ZIKV plus FU10-treated microglia. Quantitative PCR with reverse transcription, RT-qPCR; Zika virus, ZIKV.

As metabolic processes are the most dysregulated pathways during ZIKV infection at the transcriptome level, we hypothesized that inhibition of these pathways could be exploited for therapeutic effects. To determine the role of host cell metabolism in ZIKV replication, we utilized nucleoside metabolic inhibitors because nucleoside analogs have been reported to inhibit flaviviruses by interfering with RNA synthesis, methyl transferases and thymidine synthesis pathway.^28, 29, 30^ We utilized nucleoside metabolic inhibitors flurouracil and floxuridine in our experiments. Microglia cells were treated with flurouracil or floxuridine and inoculated with ZIKV. The effect of the antimetabolites floxuridine (Figures 5A and 5B) and flurouracil (Figures 5C and 5D) on ZIKV replication was assessed 48 hpi at the RNA and protein level by RT-qPCR and immunohistochemistry, respectively. Although both nucleoside analogs affected ZIKV, but the treatment of floxuridine inhibited ZIKV more efficiently in a dose-dependent manner than flurouracil. These findings show that ZIKV replication is sensitive to nucleoside metabolic pathways revealed by transcriptomic analyses.

## DISCUSSION

In this study, we analyzed the transcriptional profiles of human microglia, fibroblast, kidney and macrophage cell lines to explore the host factors that contribute to susceptibility to ZIKV infection, viral replication and host symptomology. ZIKV expression was significantly different among the cell lines, with a notably large difference between microglial cells and THP-1 monocyte-derived cells, despite both being macrophage cell lines. A recent study analyzing the cell line susceptibility in across cell types—including placental, genitourinary, neuromuscular, retinal, respiratory and liver—and species further validate our findings. Similar to our results, the study showed that THP-1-derived macrophages are relatively resistant to viral infection when compared to HEK, HeLa and SF268 neurons.^31^

By analyzing steady-state gene expression levels in cells before ZIKV infection, we identified several antiviral response genes that may contribute to the significant difference in ZIKV expression between cell types. For example, the Toll-like receptors TLR7 and TLR8 are functionally related genes and are highly expressed in THP-1-derived macrophages compared with microglia, 293FT and BJ cells. As TLR7 and TLR8 are activated by single-stranded RNAs, they likely allow THP-1 macrophages to recognize flaviviruses and produce a more robust innate immune response.^32, 33^ Moreover, THP-1-derived macrophages express tumor necrosis factor α (TNF-α) as well as CD86, a co-stimulatory molecule that has been implicated in the early and late acute phases of dengue infection. However, it is important to note that differences in basal level gene expression between cell types does not necessarily equate to functional significance because each cell type expresses unique sets of genes. Further experiments will be required to determine which of these antiviral response genes regulate ZIKV expression.

Other flaviviruses, such as WNV, dengue, yellow fever and Japanese encephalitis viruses, have shown a remarkable ability to evade the innate and adaptive immune systems.^34^ Complement proteins recognize target pathogens and act as opsonins to promote recruitment of phagocytes and lysis of infected cells. Previous studies have shown that the complement system can be compromised by the flavivirus nonstructural protein NS1, which interacts with the complement regulatory glycoprotein factor H.^35^ In addition, flaviviruses are able to evade the antibody and cellular immune response by affecting antigen presentation. The error-prone nature of flavivirus RNA polymerases leads to the accumulation of mutations and subsequent alterations in viral proteins that may help them to escape recognition by neutralizing or inhibitory antibodies.^35^

We showed that ZIKV affects the adaptive immune response and complement cascade by modulating genes such as *IL1B*, *CD4*, *IL27RA* and *A2M*. Flaviviruses downregulate *CD4* mRNA through an ns5-dependent mechanism, thereby dysregulating both the innate and adaptive immune systems.^36^ Moreover, these findings are corroborated by a recent study analyzing transcriptional changes in ZIKV-infected human neural stem cells in which leukocyte activation, cytokine production and defense response pathways were significantly dysregulated.^37^ In addition, *IL1B* has previously been linked to WNV. IL-1β is present at increased levels in the plasma and cortical neurons of WNV patients, and it plays a key role in restricting virus replication.^38^ IL-1β acts in concert with type I interferon (IFN) and the NLRP3 inflammasome to restrict WNV replication. Interestingly, *IL1B* expression was upregulated by ZIKV in THP-1-derived macrophages. But downregulated in microglia cells, which is consistent with our finding that viral replication is higher in the microglial-derived than in the monocyte-derived macrophage. These data imply that, although ZIKV is actively targeted by the innate and adaptive immune responses via monocyte-derived macrophages, it is able to effectively evade the microglial immune response once it passes through the blood–brain barrier and reaches the central nervous system.

Endothelial cells are one of the cell types infected by dengue virus, also a flavivirus, and the breakdown in endothelial barrier function causes the vascular leakage associated with hemorrhagic fever. Dengue virus type 2 suppresses TNF-α-mediated hyperpermeability and angiogenesis by modulating type I IFN.^39^ Cases of thrombocytopenia and subcutaneous bleeding have been observed in Zika patients.^40^ Our data suggest that ZIKV may affect angiogenesis and endothelial cell integrity. These findings may provide insights into the molecular mechanisms by which ZIKV passes through the blood–brain barrier.

Last, we identified a large number of differentially expressed genes associated with cellular metabolic processes following ZIKV infection. Previous studies have drawn attention to the role of virus-mediated reprogramming of host metabolic processes in pathogenesis. In accordance with our findings, transcriptional alterations in human neural stem cells inoculated with MR766 strain ZIKV revealed significant remodeling of nucleic acid metabolic processes and macromolecule biosynthesis.^37^ RNA viruses such as influenza and dengue alter fatty acid synthesis and induce glycolysis to promote viral replication, late gene synthesis and virion assembly.^25^ The role of dengue NS3 recruitment of fatty acid synthase to sites of viral replication has been dissected using RNA interference and small-molecule inhibitors in an effort to identify potential therapeutic targets.^41^ Many other viruses redistribute host cell resources to promote viral replication by altering the localization of lipids, as seen with dengue. In addition, viral infection alters the rate of host RNA metabolism to enhance the availability of nucleotides.^27^

The molecular mechanisms by which viruses redirect cellular resources and exploit the host metabolic machinery are largely unknown. Recent work showed that the adenovirus gene element E4ORF1 upregulates glucose metabolism by altering the epigenetic landscape. E4ORF1 interacts with MYC to enhance transcription of glycolytic enzymes and nucleotide biosynthesis.^42^ Here we identified several genes associated with metabolic regulation that are differentially expressed between cell types following ZIKV infection, including *CREB5*, *TERT*, *CNIH1*, *ADAM12* and *USE1*. One gene that ZIKV may exploit to regulate the host cell is *CREB5*. CREB5 (CAMP-responsive element-binding protein 5) is a transcription factor that regulates nucleotide and nucleic acid metabolism, transcription, and signal transduction, and is also highly upregulated in patients with HIV encephalitis and vaccinia virus infections.^43, 44^ Because cellular metabolism is often a limiting factor in viral replication, nucleoside/nucleotide-based therapeutics have been developed against a variety of viruses, including HIV, HBV, HCV, HSV and VZV.^26^ Indeed, nucleoside analogs including floxuridine, also known to be effective against other flaviviruses such as dengue, displayed dose-dependent inhibition of ZIKV replication.^28, 29, 30^ Further mechanistic studies will be required to gain a better understanding of how ZIKV hijacks the host cell metabolic machinery and to aid in the development of ZIKV-targeted therapeutics.

## CONCLUSIONS

ZIKV infection is an emerging disease associated with increased incidence of neurological disorders including congenital microcephaly and Guillain–Barré syndrome. Here we analyzed the transcriptomic changes associated with ZIKV infection across multiple cell types to identify novel therapeutic targets and understand the host–pathogen interaction for sustained ZIKV replication. The response to ZIKV is cell-type specific with the greatest replication found in microglia cells. ZIKV is highly expressed in microglia and downregulates immune response genes, whereas high expression of viral response genes in macrophages confers ZIKV resistance. In addition, ZIKV reprograms the host metabolic processes to enhance virus replication through the upregulation of glycolysis and RNA metabolism related genes. Antimetabolites floxuridine abrogated ZIKV replication through inhibition of host nucleoside metabolic pathways. These results reveal that thymidine synthesis pathway can be exploited to develop novel therapeutics to treat ZIKV infections.
