# Supplementary material for: Zika virus infection reprograms global transcription of host cells to allow sustained infection
Source: Emerg Microbes Infect. 2017 Apr 26;6(4):e24–. doi: 10.1038/emi.2017.9 (PMC5457678; doi:10.1038/emi.2017.9)
Supplement: Supplementary Figure S1 [file emi20179x1.pdf]

Figure S1

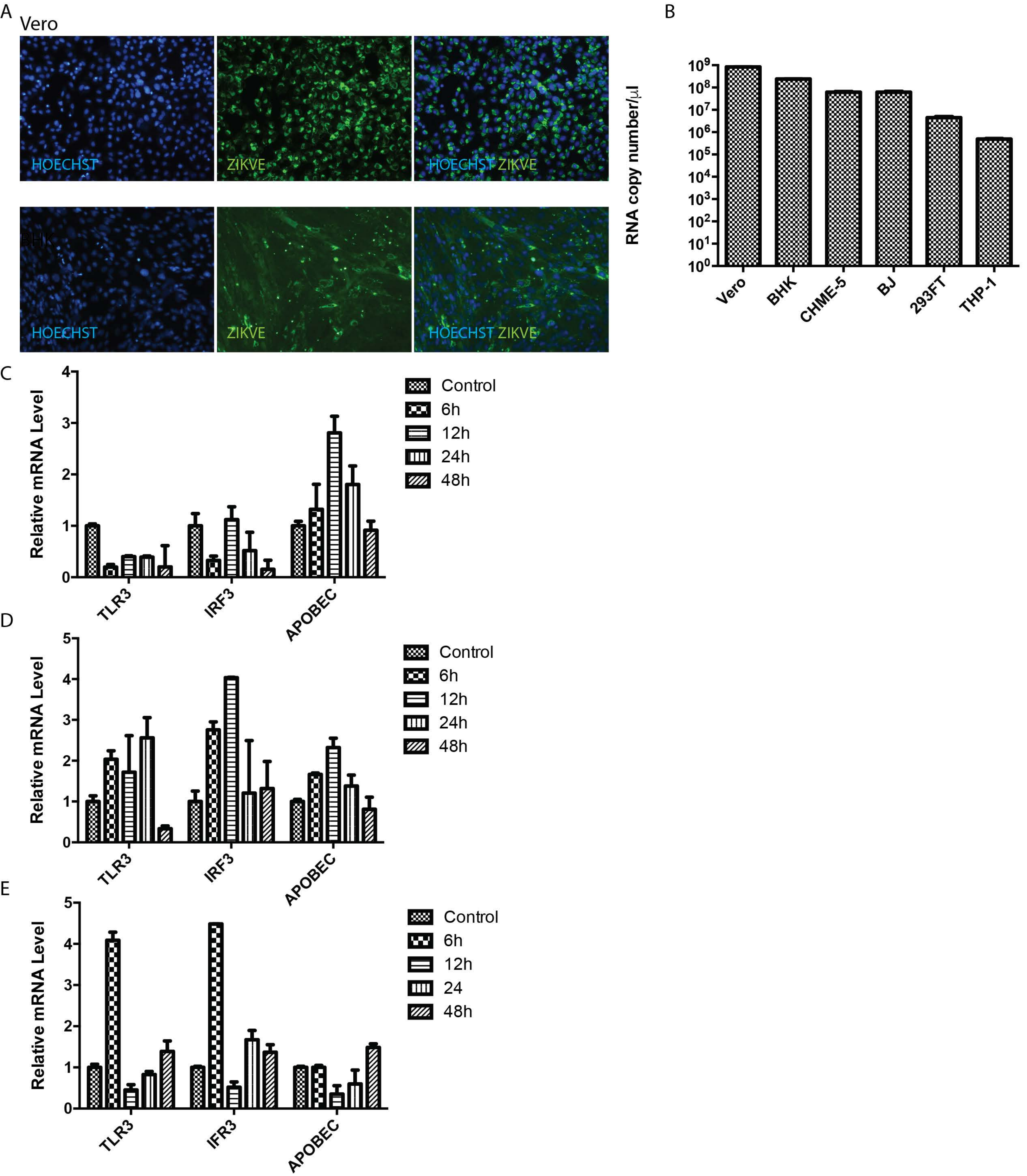
A
Vero
HOECHST
ZIKVE
HOECHST ZIKVE
HOECHST
ZIKVE
HOECHST ZIKVE
B
RNA copy number/μl
10^9
10^8
10^7
10^6
10^5
10^4
10^3
10^2
10^1
10^0
Vero
BHK
CHME-5
BJ
293FT
THP-1
C
Relative mRNA Level
4
3
2
1
0
TLR3
IRF3
APOBEC
Control
6h
12h
24h
48h
D
Relative mRNA Level
5
4
3
2
1
0
TLR3
IRF3
APOBEC
Control
6h
12h
24h
48h
E
Relative mRNA Level
5
4
3
2
1
0
TLR3
IFR3
APOBEC
Control
6h
12h
24
48h

Figure S1 Amplification of MR766 ZIKV in Vero and BHK Cells, Related to Figure

(A) Immunohistochemical staining for ZIKV expression in Vero and BHK cells. (B) RT-qPCR analysis of Vero, BHK, microglia, BJ, 293T, and THP-1 cell supernatants. (C-E) RT-qPCR analysis of innate immune response genes *TLR3*, *IRF3*, *APOBEC* in microglia, BJ and THP-1 derived macrophages, respectively.
