# Supplementary material for: Zika virus infection reprograms global transcription of host cells to allow sustained infection
Source: Emerg Microbes Infect. 2017 Apr 26;6(4):e24–. doi: 10.1038/emi.2017.9 (PMC5457678; doi:10.1038/emi.2017.9)
Supplement: Supplementary Figure S2 [file emi20179x2.pdf]

Figure S2

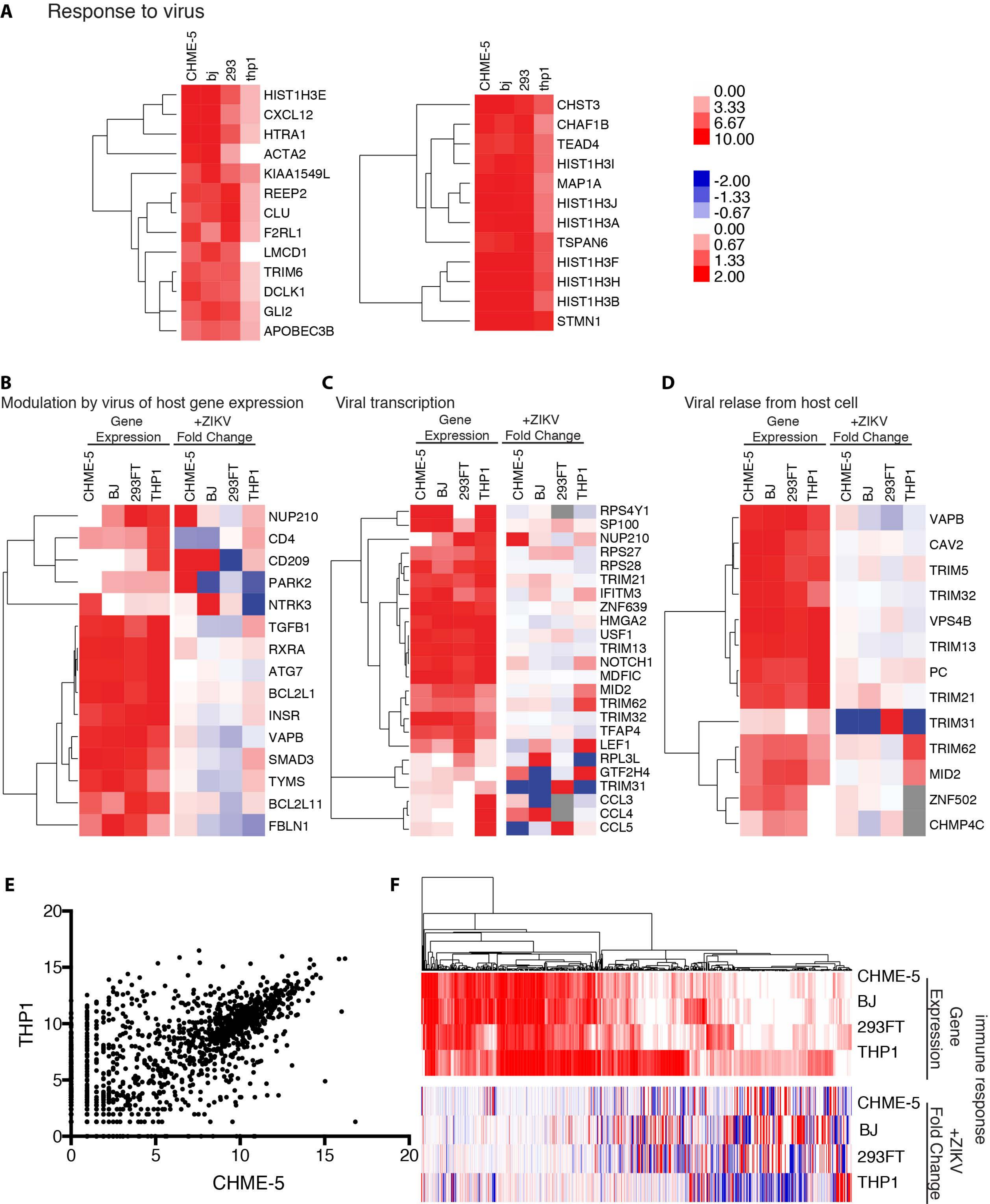

A
Response to virus
CHME-5
bj
293
thp1
HIST1H3E
CXCL12
HTRA1
ACTA2
KIAA1549L
REEP2
CLU
F2RL1
LMCD1
TRIM6
DCLK1
GLI2
APOBEC3B
CHST3
CHAF1B
TEAD4
HIST1H3I
MAP1A
HIST1H3J
HIST1H3A
TSPAN6
HIST1H3F
HIST1H3H
HIST1H3B
STMN1
0.00
3.33
6.67
10.00
-2.00
-1.33
-0.67
0.00
0.67
1.33
2.00
B
Modulation by virus of host gene expression
Gene Expression
+ZIKV Fold Change
CHME-5
BJ
293FT
THP1
NUP210
CD4
CD209
PARK2
NTRK3
TGFB1
RXRA
ATG7
BCL2L1
INSR
VAPB
SMAD3
TYMS
BCL2L11
FBLN1
C
Viral transcription
RPS4Y1
SP100
NUP210
RPS27
RPS28
TRIM21
IFITM3
ZNF639
HMGA2
USF1
TRIM13
NOTCH1
MDFIC
MID2
TRIM62
TRIM32
TFAP4
LEF1
RPL3L
GTF2H4
TRIM31
CCL3
CCL4
CCL5
D
Viral relase from host cell
VAPB
CAV2
TRIM5
TRIM32
VPS4B
TRIM13
PC
TRIM21
TRIM31
TRIM62
MID2
ZNF502
CHMP4C
E
THP1
CHME-5
0
5
10
15
20
F
CHME-5
BJ
293FT
THP1
Gene Expression
+ZIKV Fold Change
immune response

Figure S2 Analysis of Viral Response and Immune-Related Genes Reveals Potential Antiviral Targets, Related to Figure 3

(A) Heat map of genes highly expressed in microglia, BJ, and 293T cells, but not in THP-1 derived macrophages, from the analysis of “response to virus”-associated genes in Figure 3A. Left scale bar: 0:10 represents gene expression log2(RPKM+1) for all cell times. Right scale bar: -2:2 represents fold change in gene expressed between mock treated and ZIKV infected cells.

(B–D) Hierarchical clustering of differentially expressed genes related to “modulation by virus of host gene expression” (B), “viral transcription” (C), and “viral release from host cell” (D) between mock-infected cells (left) and their associated fold change in expression after ZIKV infection (right).

(E) Scatterplot of immune response genes showing higher expression of immune-related genes in THP-1 derived macrophages compared with microglia cells.

(F) Hierarchical clustering of differentially expressed genes related to “immune response” in mock-infected cells (top) and their associated fold change in expression after ZIKV infection (bottom). Also see Table 2.
