# Supplementary material for: Zika virus infection reprograms global transcription of host cells to allow sustained infection
Source: Emerg Microbes Infect. 2017 Apr 26;6(4):e24–. doi: 10.1038/emi.2017.9 (PMC5457678; doi:10.1038/emi.2017.9)
Supplement: Supplementary Figure S3 [file emi20179x3.pdf]

Figure S3

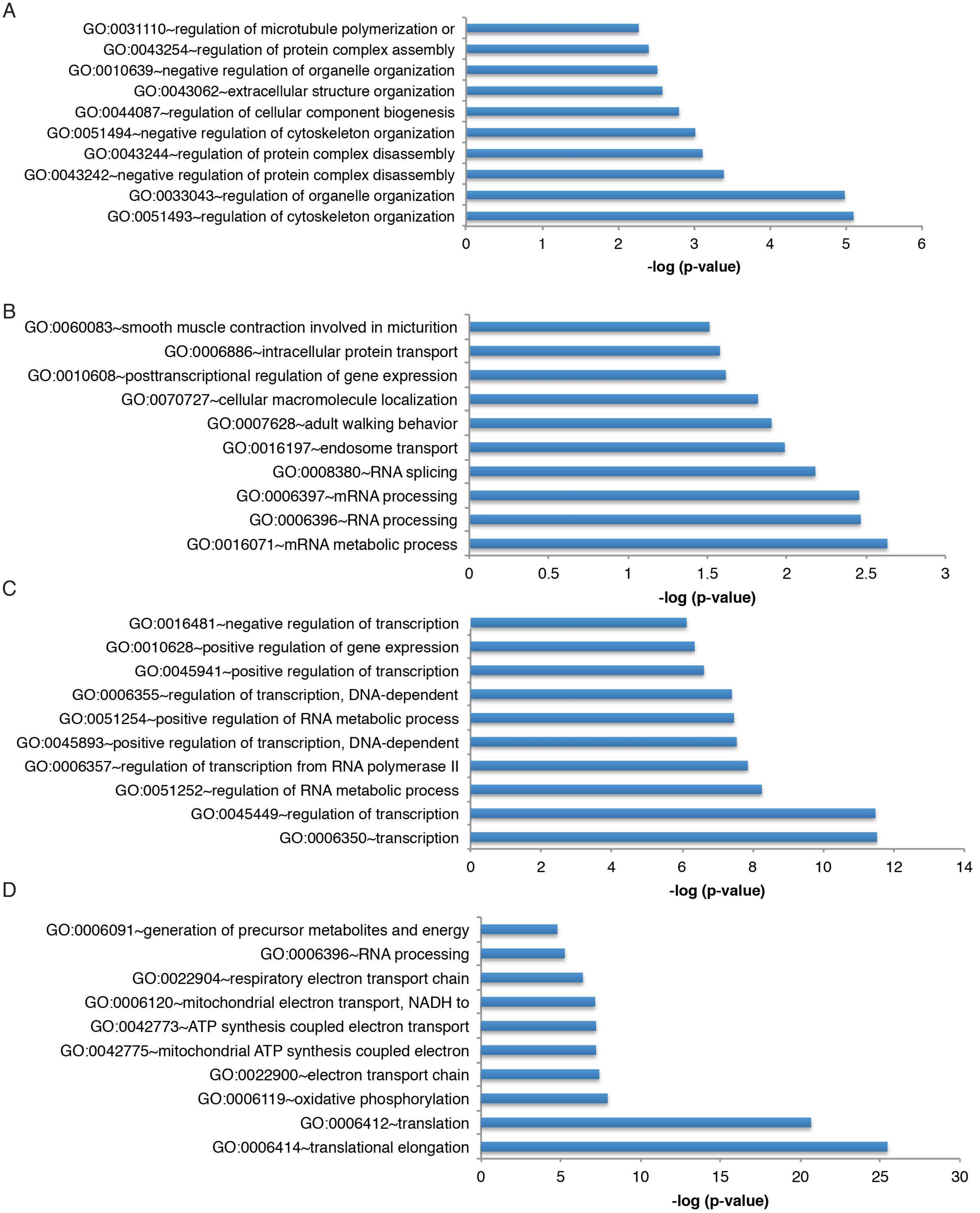
A
GO:0031110~regulation of microtubule polymerization or
GO:0043254~regulation of protein complex assembly
GO:0010639~negative regulation of organelle organization
GO:0043062~extracellular structure organization
GO:0044087~regulation of cellular component biogenesis
GO:0051494~negative regulation of cytoskeleton organization
GO:0043244~regulation of protein complex disassembly
GO:0043242~negative regulation of protein complex disassembly
GO:0033043~regulation of organelle organization
GO:0051493~regulation of cytoskeleton organization
0 1 2 3 4 5 6
-log (p-value)
B
GO:0060083~smooth muscle contraction involved in micturition
GO:0006886~intracellular protein transport
GO:0010608~posttranscriptional regulation of gene expression
GO:0070727~cellular macromolecule localization
GO:0007628~adult walking behavior
GO:0016197~endosome transport
GO:0008380~RNA splicing
GO:0006397~mRNA processing
GO:0006396~RNA processing
GO:0016071~mRNA metabolic process
0 0.5 1 1.5 2 2.5 3
-log (p-value)
C
GO:0016481~negative regulation of transcription
GO:0010628~positive regulation of gene expression
GO:0045941~positive regulation of transcription
GO:0006355~regulation of transcription, DNA-dependent
GO:0051254~positive regulation of RNA metabolic process
GO:0045893~positive regulation of transcription, DNA-dependent
GO:0006357~regulation of transcription from RNA polymerase II
GO:0051252~regulation of RNA metabolic process
GO:0045449~regulation of transcription
GO:0006350~transcription
0 2 4 6 8 10 12 14
-log (p-value)
D
GO:0006091~generation of precursor metabolites and energy
GO:0006396~RNA processing
GO:0022904~respiratory electron transport chain
GO:0006120~mitochondrial electron transport, NADH to
GO:0042773~ATP synthesis coupled electron transport
GO:0042775~mitochondrial ATP synthesis coupled electron
GO:0022900~electron transport chain
GO:0006119~oxidative phosphorylation
GO:0006412~translation
GO:0006414~translational elongation
0 5 10 15 20 25 30
-log (p-value)

Figure S3 Gene ontology analysis of differentially expressed genes in microglia (A), BJ (B), 293FT (C), and THP-1 (D) cells, Related to Figure 4
